# Results of integrating recency testing into routine index testing services in Lusaka, Zambia: A mixed effects regression analysis

**DOI:** 10.1371/journal.pone.0361598

**Published:** 2026-10-30

**Authors:** Jake M. Pry, Shilpa S. Iyer, Chilambwe Mwila, Christiana Frimpong, Misinzo Moono, Musunge Mulabe, Mirriam Nanyangwe, Herbert Kapesa, Lumbani Phiri, Patricia Maritim, Melissa M. Arons, Tiffiany M. Aholou, Carolyn Bolton Moore, Theodora Savory-van Huis, Michael E. Herce

**Affiliations:** 1 Centre for Infectious Disease Research in Zambia (CIDRZ), Lusaka, Zambia; 2 Division of Epidemiology, School of Medicine, University of California, Davis, California, United States of America; 3 Global Vaccine Preventable Diseases Laboratory Networks, World Health Organization, Geneva, Switzerland; 4 Division of Global HIV and TB, Global Health Center, U.S. Centers for Disease Control and Prevention, Atlanta, Georgia, United States of America; 5 School of Medicine, University of Alabama, Birmingham, Alabama, United States of America; 6 Institute for Global Health & Infectious Diseases, University of North Carolina, Chapel Hill, North Carolina, United States of America; Dr D Y Patil Vidyapeeth University Dr D Y Patil Medical College Hospital and Research Centre: Dr D Y Patil Medical College Hospital and Research Centre, INDIA

## Abstract

**Introduction:**

As countries move toward epidemic control, reaching PLHIV unaware of their status becomes increasingly difficult, necessitating targeted strategies like index testing. We evaluated a strategy incorporating identification of recent HIV acquisition into routine index testing in Zambia.

**Methods:**

Consecutive adults newly diagnosed with HIV at two Lusaka health facilities were enrolled into a pilot interventional cohort to undergo rapid test for recent infection (RTRI) and viral load testing per the national recent infection testing algorithm (RITA). Consenting participants received RITA results; those with recent infection were offered additional partner elicitation, assisted partner notification, and contact tracing after routine index testing services. We compared test positivity between contacts of index participants with long-term versus recent HIV acquisition and used regression and latent class analyses to identify factors associated with recent acquisition.

**Results:**

Between May 2021–March 2022, we enrolled 321 of 393 (81.7%) newly diagnosed PLHIV and analyzed 317, with median age 31 years (IQR: 25–37) and most (63.1%) female. HIV positivity did not differ between contacts of RITA-recent (28.6%) versus long-term (27.9%) index participants (p = 0.942). Recency was strongly associated with condomless sex at last sex (PR: 7.25, 95% CI: 3.06–17.19), which latent class analysis confirmed was consistently associated with other high-risk behaviors. Of 305 participants completing a social harms survey, 27 (8.9%) reported ≥1 harm attributed to recency testing.

**Conclusion:**

While integrating recency testing into routine index testing was feasible, our findings do not support its use to improve efficiency. Risk behavior assessment may help prioritize PLHIV for recency and index testing.

## Introduction

Despite dramatic progress toward ensuring universal access to antiretroviral therapy (ART) for people living with HIV (PLHIV) in Zambia, new approaches are needed to close the HIV diagnosis gap such that 95% of all PLHIV know their HIV status [1]. Index testing is a proven strategy for reaching PLHIV unaware of their status that relies on testing the sexual and needle-sharing contacts and biological children of PLHIV. Although HIV index testing has been shown to be effective and has been scaled up in more than 20 countries, including Zambia [2], it is a time and resource-intensive strategy. Therefore, targeted strategies are needed to more efficiently and sustainably deliver HIV testing services (HTS) to populations at high risk for HIV acquisition.

Prioritizing HIV case-finding based on differential risk has been the conceptual foundation of index testing and other proven HIV testing approaches [3–5]. As a result, the World Health Organization (WHO) recommends HIV risk-based screening tools to screen-in persons with high-risk behaviors to receive provider-initiated testing and counseling [6]. Certain risk behaviors, including no condom use at last sex, participation in commercial sex, and alcohol consumption have previously been shown to be associated with increased risk of HIV acquisition [7,8].

Since 2011, the WHO has provided guidance on using HIV recency testing to identify geographic areas of increased HIV transmission [9,10]. Tests for recent infection (TRI) identify biomarkers that signal HIV acquisition within a particular time frame (six-month mean duration of infection) when PLHIV may be at comparably higher risk for transmitting HIV to their sexual and/or needle-sharing partner(s) [11–13]. Although recency testing for HIV has been used primarily at population level, increasingly, it has been considered as a potential approach for identifying groups at the greatest risk of HIV acquisition who may benefit from more targeted HIV case finding and prevention [14–17]. In theory, recency testing could be used to prioritize index clients with recent HIV infection for enhanced sexual partner elicitation and prioritized contact counseling and tracing. For example, an HIV prevention project in Greece found five times greater HIV positivity among contacts with recently acquired HIV compared to contacts of index clients with long-term infection [18].

With support from the United States President’s Emergency Plan For AIDS Relief (PEPFAR), Zambia developed a national recent infection testing algorithm (RITA) employing rapid tests for recent infection (RTRI) and quantitative HIV viral load (VL) to help the national HIV program identify, monitor, and respond to recent HIV infections among newly-diagnosed PLHIV [19]. While the use of recency testing holds promise as a tool to enable precision public health at facility, sub-national, and national levels, its effects when used at an *individual* level to potentially enable more efficient social network-based HIV testing are unknown. Moreover, it is unclear if integrating recency testing into index testing offers any advantage over mainstay strategies that prioritize populations based on reported HIV risk behaviors. Finally, the associations between behavioral risk factors and recent HIV acquisition is largely unexplored, and may be key to optimizing current HIV testing approaches to reach the most high-risk groups with HIV testing services in pursuit of global and national 95-95-95 targets [20].

We conducted a pilot study integrating HIV recency testing into routine index testing services to prioritize index clients with recently acquired HIV for partner elicitation, assisted partner notification (APN), and tracing and testing of their contacts. As part of the study, we evaluated the effects of the recency testing strategy on contact positivity according to index recency status (i.e., recent versus long-term HIV acquisition) with the expectation that contact(s) of individuals identified as having recently acquired HIV may be at higher risk of HIV, and thus, have different potential index testing positivity. Additionally, we characterized the HIV acquisition risk profiles of the populations reached and the potential social harms of employing this integrated approach. In this paper, we report results from the pilot to examine how, if at all, recency results can be used to optimize existing HIV index testing programs.

## Methods

### Design overview

We conducted a pilot study called “Recency Enhanced Algorithm for Controlling HIV in Zambia (REACHZ)” using convergent mixed methods to assess integrating recency testing within Lusaka’s PEPFAR-supported, Ministry of Health (MOH)-implemented routine index testing program. This “enhanced” index testing (eIT) approach aims to prioritize individuals potentially more likely to acquire and transmit HIV. Focusing on the quantitative REACHZ data, we evaluated a cohort of individuals presenting for routine HIV testing and found to be living with HIV and not previously aware of their positive HIV status in Lusaka, Zambia.

### Study site and population

The study was conducted at two high-volume U.S. Centers for Disease Control and Prevention (CDC)/PEPFAR-supported MOH health facilities and their surrounding communities (i.e., “sites”) in urban Lusaka district, Zambia. The two sites were purposively selected to leverage existing CDC/PEPFAR-funded index testing programming, infrastructure, and investments supporting index testing delivery according to the PEPFAR minimum standards for safe and ethical index testing [21]. Both study sites have implemented the MOH’s HIV recency surveillance program since 2019.

The source population was newly diagnosed adult PLHIV offered index testing at these sites, including members of general and priority populations such as young men, adolescent girls and young women, and mobile populations. We excluded known high-risk populations (e.g., female sex workers, men who have sex with men) and venues serving them due to heightened social harm concerns.

### Eligibility criteria

Individuals were eligible if aged ≥18 years, newly diagnosed with HIV via the national testing algorithm from 1 May 2021 to 30 March 2022 at one of two participating study sites, able to consent, and willing to provide locator information. Written informed consent was offered in English, Nyanja, and Bemba.

### Standard of care index testing services

Newly diagnosed patients are linked to care and offered voluntary index testing. For consenting individuals, healthcare workers elicit sexual and needle-sharing partners through an eight-step process: 1) introduce index testing services, 2) obtain consent and offer assisted partner notification, 3) elicit partners and biological children under 19 with unknown HIV status, 4) screen for intimate partner violence, 5) identify preferred notification methods, 6) contact partners using preferred approaches, 7) record testing outcomes, and 8) provide appropriate services based on HIV status [21]. National guidelines recommend contact tracing and HIV testing services referral for all reported partners of individuals newly diagnosed with HIV [22]. While subsequent rounds of contact elicitation are recommended at each encounter with HIV care, they rarely happen in routine practice. These procedures were documented routinely in MOH paper-based records, which were securely stored at the sites.

### Pilot intervention

This pilot assessed integrating point-of-care HIV recency testing into routine index services using the Asanté™ HIV-1 Rapid Recency® Assay (Sedia Biosciences, Portland, Oregon, USA) done by trained site health care workers (HCWs). Participants with a new, positive HIV diagnostic test result received rapid recency testing during their first care visit, with confirmatory lab testing to determine RTRI recent or RTRI long-term HIV status. Recency results were returned at the first follow-up visit 7–14 days later alongside routine baseline test results. A re-elicitation of sexual partners was also conducted at the next follow-up visit among those found to be RITA-recent.

### Laboratory procedures

Individuals who presented to the study sites for routine HTS were tested by HCWs according to the national HIV testing algorithm (i.e., Determine1/2 and Bioline) [23,24]. The Asanté™ RTRI helps distinguish recent from long-term HIV acquisition [25], and was performed at the point of care in the health facility by non-laboratory healthcare workers trained by the study staff according to manufacturer specifications. VLs were quantified for all samples with a recent RTRI result and a subset with long-term results using the Aptima® HIV-1 Quant Assay (Hologic® Panther platform). At the CIDRZ central laboratory, a trained technician performed a repeat RTRI, VL test, and quality control procedures. Combined Asanté™ RTRI and VL results were used to classify samples per national RITA definitions [23,24]. Participants were classified as ‘RITA-recent’ if they reported no prior HIV diagnosis or ART exposure, had a recent Asanté™ RTRI result, and VL ≥ 1,000 copies/ml. All others—including those with a long-term RTRI irrespective of VL, or a recent RTRI with VL < 1,000 copies/ml—were classified as ‘RITA long-term.’ Results were returned to participants at their next facility visit, approximately 7–14 days after enrolment.

### Study procedures

Those diagnosed with HIV were sensitized to the REACHZ study by trained staff. Interested individuals were referred for informed consent and recency testing. During enrolment, staff administered a 40-item bio-behavioral survey and locator form via interview to facilitate follow-up and ensure provision of recency test results. After returning CIDRZ RITA results at the first follow-up visit, staff implemented, with participant consent, a second round of contact elicitation for participants classified with recently acquired HIV (with the logic that participants may be more likely to report additional contacts upon learning their recency status). Additional contacts were provided to the MOH index testing program to support a second round of contact tracing per national guidelines. Index contacts were categorized as known HIV-positive, unknown HIV status and offered testing, declined testing, or could not be traced. A second study-specific follow-up visit was scheduled ~90 days after enrolment to assess for possible social harm, with staff referring affected individuals for services per MOH guidelines [23,24].

### Data collection

We administered a bio-behavioral survey (**S1 File Form**), at enrollment, and a separate 19-item semi-structured survey (**S2 File** **Form**) exploring potential instances and causes of social harm at the second study follow-up visit. Surveys were administered using a dedicated paper-based case reporting form (CRF) stored securely in study facilities and accessible only to trained study staff. All index testing outcomes, including for the second round of contact elicitation directed by recency testing and the outcomes of all rounds of contact tracing and testing, were documented using standard MOH tools. Study staff abstracted existing health information from these routine data sources for analysis. All study-specific CRFs and abstracted index testing program data were entered into a secure, password-protected District Health Information System 2 (DHIS2) database stored on a secure CIDRZ server.

### Analysis

We summarized index testing cascades using counts, proportions, and descriptive statistics. We used mixed effects Poisson regression to estimate prevalence of recency by participant sociodemographic and behavioral characteristics allowing for random effects at the facility level to account for potential facility level correlation. We conducted a latent class analysis among variables associated with HIV acquisition to identify behavioral patterns that might be associated with RITA-recent classification [26,27]. This analysis considered variables of alcohol consumption, no condom use at last sex, transactional sex, and ever having sex while drunk or high on drugs. The number of latent classes was decided in the context of data limitations and the ability to detect meaningful differences across classes. Additionally, we calculated and illustrated marginal probabilities for the identified biobehavioral profiles. All analyses were conducted using Stata 18 (StataCorp LP, College Station, Texas, USA).

### Ethics

The study protocol was approved by ethical review bodies of the University of Zambia (#1157−2020), the Zambian National Health Research Authority, the U.S. CDC Global Health Center (#CGH-ZAM-12/18/20-5d5a1), and the University of Alabama at Birmingham (#300006066). All participants provided written informed consent.

## Results

### Overview

A total of 393 people newly diagnosed with HIV at the two study sites were invited for study participation, of whom 344 (87.5%) were found eligible, 322 (81.9%) consented, 321 (81.7%) completed enrollment procedures, and 317 (80.7%) had requisite information to classify per the completed RITA algorithm (Fig 1).

**Fig 1:**
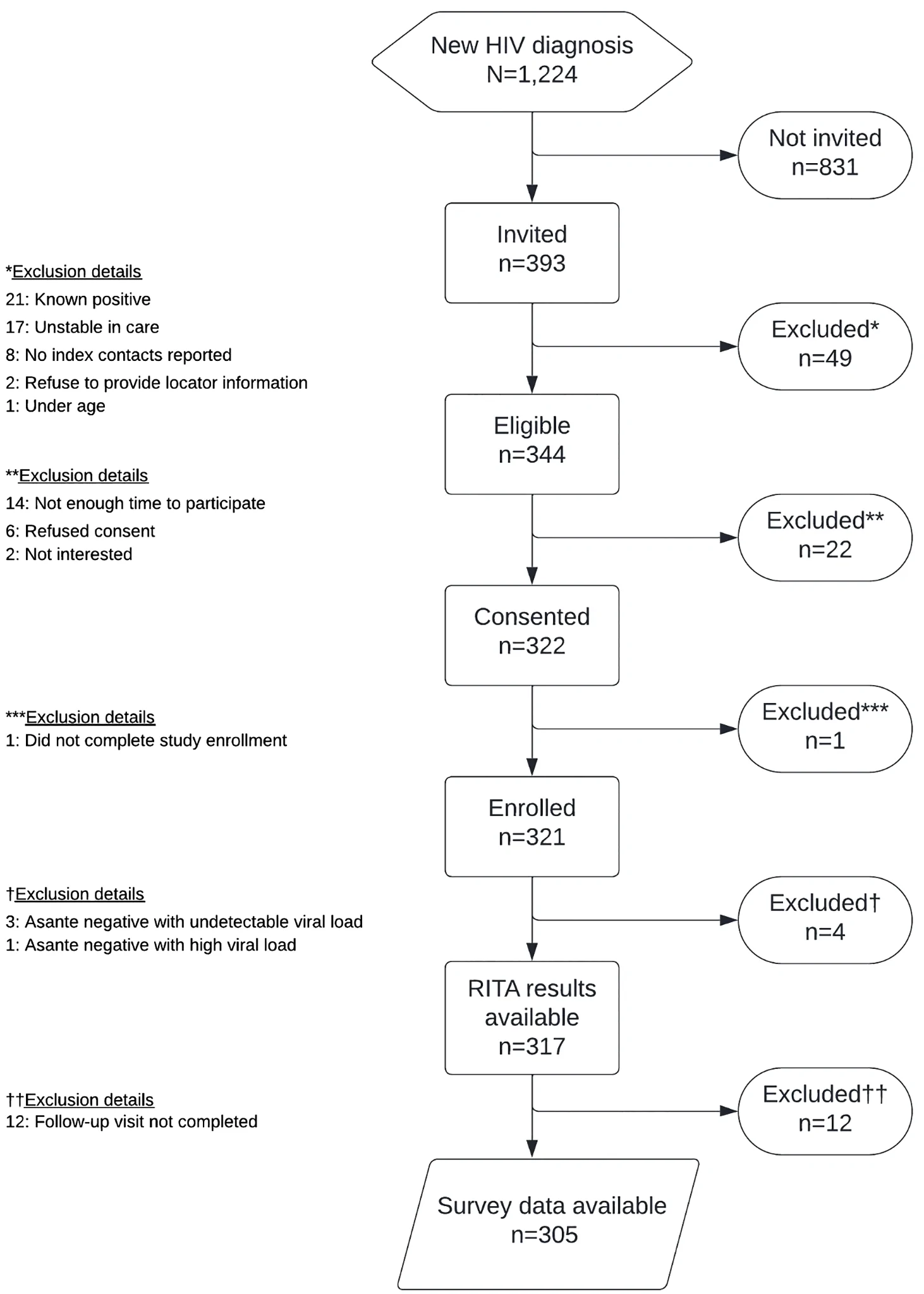
Participant flow diagram.

Of the 317 newly HIV-positive participants with available RITA results, 20 (6.3%, 95% confidence interval [CI]:3.9, 9.6%) were classified as RITA recent and 297 were classified as RITA long-term (93.7%, 95% CI:90.4, 96.1%). The majority (63.2%) of participants were female with median age 30 years (interquartile range [IQR]:25–37 years) (**Table 1**).

**Table 1:** Characteristics of participants by recent infection testing algorithm (RITA) classification (N = 317).

*Factor*	*Level*	*Total*	*RITA*	*RITA*	*p-value*
			*Recent*	*Long-term*	
		*n (%)*	*n (%)*	*n (%)*	
**N**		317	20 (6.3)	297 (93.7)	
**Birth sex**	Female	200 (63.1)	14 (70.0)	186 (62.6)	0.508
	Male	117 (36.9)	6 (30.0)	111 (37.4)	
**Age**	Median (IQR)	30 (26–37)	28.5 (24–37)	31 (26–37)	0.477
	18-24 years	67 (21.1)	6 (30.0)	61 (20.5)	0.732
	25-34 years	138 (43.5)	7 (35.0)	131 (44.1)	
	35-44 years	90 (28.4)	6 (30.0)	84 (28.3)	
	45 + years	22 (6.9)	1 (5.0)	21 (7.1)	
**Marital status**	Single	97 (30.6)	8 (40.0)	89 (30.0)	0.597
	Married	158 (49.8)	7 (35.0)	151 (50.8)	
	Divorced/Separated	50 (15.8)	4 (20.0)	46 (15.5)	
	Widowed	12 (3.8)	1 (5.0)	11 (3.7)	
**Consent language**	English	94 (29.7)	5 (25.0)	89 (30.0)	0.215
	Nyanja	160 (50.5)	8 (40.0)	152 (51.2)	
	Bemba	63 (19.9)	7 (35.0)	56 (18.9)	
**Educational attainment**	Less than secondary	94 (29.7)	4 (20.0)	90 (30.3)	0.557
	Secondary	202 (63.7)	14 (70.0)	188 (63.3)	
	College/ University	21 (6.6)	2 (10.0)	19 (6.4)	
**Employment status**	Not working/ Unemployed	100 (31.5)	9 (45.0)	91 (30.6)	0.165
	Employed/	63 (19.9)	2 (10.0)	61 (20.5)	
	Working				
	Self-employed	139 (43.8)	7 (35.0)	132 (44.4)	
	Student	8 (2.5)	2 (10.0)	6 (2.0)	
	Retired	3 (0.9)	0 (0.0)	3 (1.0)	
	Other	4 (1.3)	0 (0.0)	4 (1.3)	
**Has a mobile phone**	No	49 (15.5)	1 (5.0)	48 (16.2)	0.181
	Yes	268 (84.5)	19 (95.0)	249 (83.8)	
**Tested for HIV previously**	No	54 (17.0)	1 (5.0)	53 (17.8)	0.280
	Yes	259 (81.7)	19 (95.0)	240 (80.8)	
	Don’t know	4 (1.3)	0 (0.0)	4 (1.3)	
**Facility**	A	118 (37.2)	11 (55.0)	107 (36.0)	0.089
	B	199 (62.8)	9 (45.0)	190 (64.0)	
**Month of enrollment**	May-21	12 (3.8)	0 (0.0)	12 (4.0)	0.841
	Jun-21	48 (15.1)	2 (10.0)	46 (15.5)	
	Jul-21*	0 (0.0)	0 (0.0)	0 (0.0)	
	Aug-21	31 (9.8)	3 (15.0)	28 (9.4)	
	Sep-21	65 (20.5)	6 (30.0)	59 (19.9)	
	Oct-21	35 (11.0)	3 (15.0)	32 (10.8)	
	Nov-21	21 (6.6)	1 (5.0)	20 (6.7)	
	Dec-21	12 (3.8)	0 (0.0)	12 (4.0)	
	Jan-22	48 (15.1)	3 (15.0)	45 (15.2)	
	Feb-22	45 (14.2)	2 (10.0)	43 (14.5)	

Note: IQR – interquartile range, categorical variable p-value according to Chi-square test, continuous variable (median age) p-value according to Wilcoxon rank-sum test.

*Enrollment for July 2021 was stopped due to increased COVID-19 cases from the delta wave in Zambia.

### Index testing metrics

Among 20 RITA-recent index participants, 53 contacts were elicited after two rounds of elicitation (elicitation ratio: 2.65) (**Table 1**). Of the 53 contacts disclosed by RITA-recent participants (initial elicitation: n = 39; re-elicitation: n = 14), 28 were tested (52.8%) and 8 (28.6%; 95% CI:10.7, 46.4%) were newly identified as living with HIV (Fig 2, **S1 Table**). The proportion of newly positive index contacts among RITA-recent participants was higher for initial elicitation contacts compared to re-elicitation contacts at 30.0% and 5.0%, respectively (**S1 Table**). Among 297 index participants with long-term infection, 479 contacts were elicited (elicitation ratio:1.61). Of these, 240 (50.1%) were tested, and 67 (27.9%; 95% CI: 22.2, 33.6%) were newly identified as HIV-positive (Fig 2).

**Fig 2:**
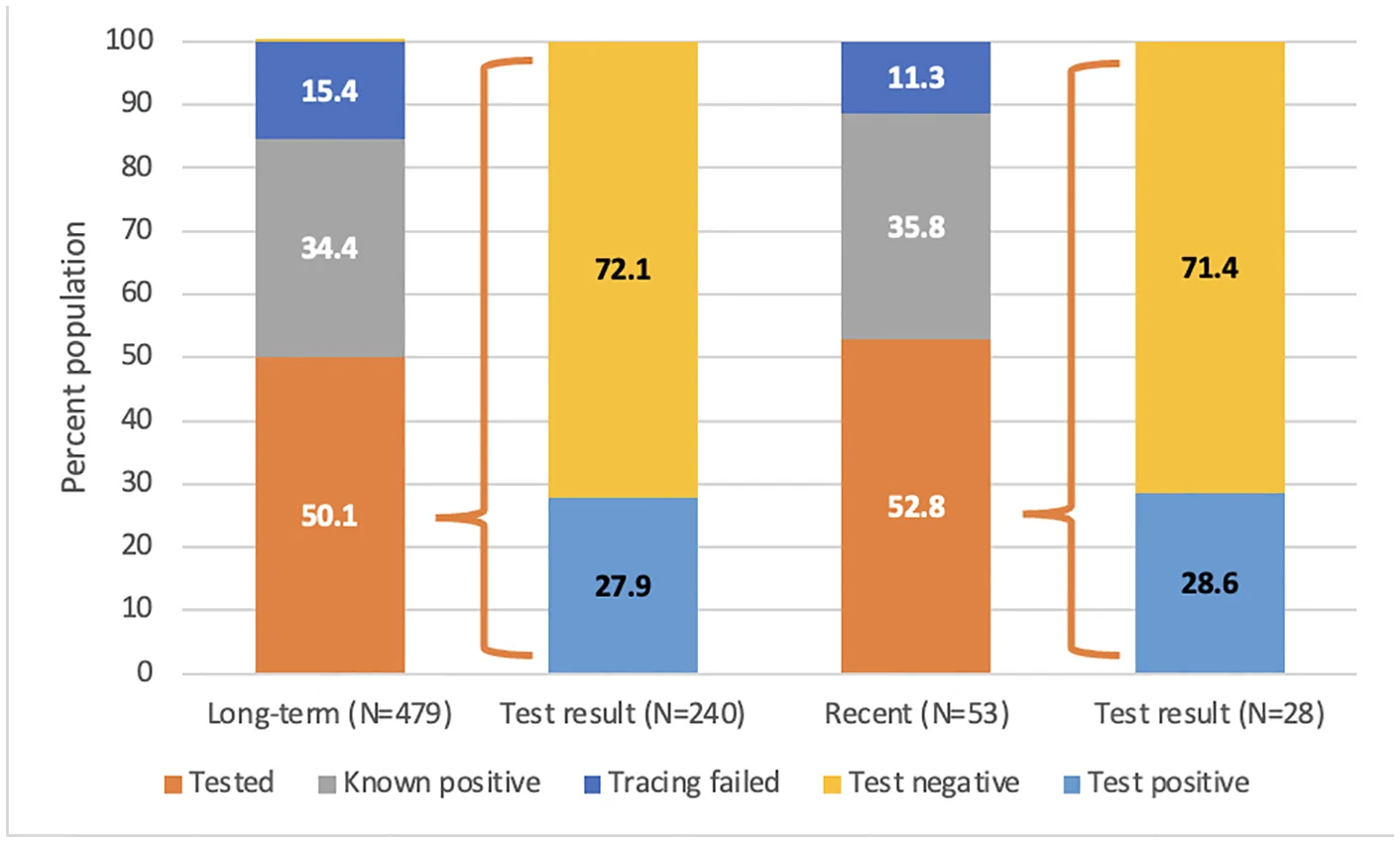
Contact tracing outcomes by index client recency status—RITA-long-term versus RITA-recent (N_contacts elicited_ = 532).

### Reported risk behaviors

Most participants reported no condom use at last sex (n = 228, 71.9%), with a higher proportion of participants with RITA-recent classification reporting no condom use at last sex (95.0%) compared to participants with long-term infection (70.4%) (**Table 2**). Similarly, a significantly higher proportion of participants with RITA-recent classification (95.0%), compared to those with long-term infection (74.1%), reported any unprotected sex in the last 12 months (p = 0.035). Despite these differences, a higher proportion of participants with RITA-recent classification perceived their HIV risk status to be “low risk” (70.0% vs 41.1%, p = 0.053).

**Table 2:** Results from bio-behavioral survey by recency status—RITA-long-term versus RITA-recent (N = 317).

*Factor*	*Level*	*Total*	*RITA*	*RITA*	*p-value*
			*Recent*	*Long-term*	
		n (%)	n (%)	n (%)	
**N**		317 (100.0)	20 (6.3)	297 (93.7)	
**Type of partner at last sexual encounter**	Regular	222 (70.0)	14 (70.0)	208 (70.0)	0.656
	Casual	77 (24.3)	4 (20.0)	73 (24.6)	
	One time	15 (4.7)	2 (10.0)	13 (4.4)	
	Sex work	3 (0.9)	0 (0.0)	3 (1.0)	
**Currently consume alcohol**	No	159 (50.2)	11 (55.0)	148 (49.8)	0.655
	Yes	158 (49.8)	9 (45.0)	149 (50.2)	
**Quantity of alcohol consumed per week**	0 drinks	159 (50.2)	11 (55.0)	148 (49.8)	0.168
	1-2 drinks	16 (5.0)	0 (0.0)	16 (5.4)	
	3-5 drinks	49 (15.5)	3 (15.0)	46 (15.5)	
	5-7 drinks	25 (7.9)	4 (20.0)	21 (7.1)	
	>7 drinks	68 (21.5)	2 (10.0)	66 (22.2)	
**Ever had sex while drunk of high**	No	187 (59.0)	11 (55.0)	176 (59.3)	0.708
	Yes	130 (41.0)	9 (45.0)	121 (40.7)	
**Condom use at last sex**	Used condom at last sex	87 (27.4)	1 (5.0)	86 (29.0)	0.060
	No condom at last sex	228 (71.9)	19 (95.0)	209 (70.4)	
	Don’t know	2 (0.6)	0 (0.0)	2 (0.7)	
**Ever used injection drugs**	No	313 (98.7)	20 (100.0)	293 (98.7)	0.601
	Yes	4 (1.3)	0 (0.0)	4 (1.3)	
**Last partner ever used injection drugs**	Do not know	33 (10.4)	4 (20.0)	29 (9.8)	0.257
	No	273 (86.1)	16 (80.0)	257 (86.5)	
	Yes	11 (3.5)	0 (0.0)	11 (3.7)	
Transactional sex in the past 12 months*	No	225 (71.0)	12 (60.0)	213 (71.7)	0.264
	Yes	92 (29.0)	8 (40.0)	84 (28.3)	
**Perceived risk of HIV**	Don’t Know	46 (14.5)	0 (0.0)	46 (15.5)	0.053
	High	79 (24.9)	3 (15.0)	76 (25.6)	
	Low	136 (42.9)	14 (70.0)	122 (41.1)	
	Medium	56 (17.7)	3 (15.0)	53 (17.8)	
**Have ever taken PrEP**	No	100 (90.9)	12 (100.0)	88 (89.8)	0.246
	Yes	10 (9.1)	0 (0.0)	10 (10.2)	
Stigmatized**	No	269 (90.0)	18 (94.7)	251 (89.6)	0.475
	Yes	30 (10.0)	1 (5.3)	29 (10.4)	
**Unprotected sex in last 12 months**	No	78 (24.6)	1 (5.0)	77 (25.9)	0.035
	Yes	239 (75.4)	19 (95.0)	220 (74.1)	
**Ever had anal sex**	No	297 (93.7)	20 (100.0)	277 (93.3)	0.487
	Yes	18 (5.7)	0 (0.0)	18 (6.1)	
	Don’t know	2 (0.6)	0 (0.0)	2 (0.7)	

Note: Note: p-value according to Chi-square test.

*Respond “Yes” to Q17 “Have you exchanged sex for money, goods or services in the last 12 months?” in S1 File Form or respond “Yes” to Q18 “Have you given money, goods or services in exchange for sex in the last 12 months” in S1 File Form.

**Respond “Yes” to Q9 “Do you feel stigmatized (ignored, shamed) by your social circle because you participated in this study?” in S1 File Form.

### Associations between risk behaviors and recent HIV infection

In unadjusted analyses, we observed statistically significant associations between recent HIV acquisition and marital status, preferred language, educational attainment, type of partner at last sexual encounter, and condom use at last sexual encounter (**Table 3**). The prevalence of recent acquisition was significantly higher for participants who reported their last sexual encounter being with a one-time partner (PR: 2.39, 95% CI: 2.03, 2.81) compared to those reporting their last sexual encounter with a regular partner. Prevalence of recent HIV acquisition among participants who reported no condom use at their last sexual encounter was significantly higher (PR: 7.25, 95% CI: 3.06, 17.19) compared to those reporting condom use (**Table 3**). Though not statistically significant, the prevalence of recent HIV acquisition was higher among those that reported having any condomless sex in the past 12 months (PR: 6.20, 95% CI: 0.46, 82.85).

**Table 3:** Unadjusted regression results for recent HIV infection (N = 317).

*Variable*	*Level*	*PR*	*95% CI*	*p-value*
Sex	Female	ref	–	–
	Male	0.73	(0.26, 2.02)	0.541
Age	18-24 years	1.36	(0.30, 6.04)	0.688
	25-34 years	0.75	(0.22, 2.61)	0.651
	35-44 years	ref	–	–
	45 + years	0.68	(0.18, 2.6)	0.574
Marital status	Married	ref	–	–
	Single	1.87	(0.48, 7.29)	0.368
	Divorced/Separated	1.79	(1.66, 1.93)	<0.001
	Widowed	1.89	(0.42, 8.51)	0.405
Consent language	English	ref	–	–
	Nyanja	0.84	(0.71, 0.99)	0.035
	Bemba	2.12	(0.41, 11.07)	0.371
Educational attainment	Less than secondary	ref	–	–
	Secondary	1.66	(0.85, 3.26)	0.137
	College/ University	2.29	(1.46, 3.60)	<0.001
Have mobile phone	No	ref	–	–
	Yes	3.47	(0.37, 32.74)	0.277
Tested for HIV before	No	ref	–	–
	Yes	3.93	(0.60, 25.8)	0.154
Currently consume alcohol	No	ref		
	Yes	0.80	(0.50, 1.29)	0.365
Quantity of alcohol consumed per week	0 drinks/do not drink	ref	–	–
	1-2 drinks	–	–	–
	3-5 drinks	0.88	(0.28, 2.78)	0.827
	5-7 drinks	2.27	(0.93, 5.53)	0.071
	>7 drinks	0.42	(0.03, 7.02)	0.545
Ever had sex while drunk or high	No	ref	–	–
	Yes	1.16	(0.81, 1.65)	0.413
Transactional sex in the past 12 months*	No	ref	–	–
	Yes	1.67	(0.62, 4.47)	0.309
Stigmatized**	No	ref	–	–
	Yes	0.48	(0.17, 1.30)	0.147
Type of partner at last sexual encounter	Regular	ref	–	–
	Casual	0.85	(0.33, 2.17)	0.731
	One time	2.39	(2.03, 2.81)	<0.001
	Sex work	n/a	n/a	n/a
Access to condoms	Yes	ref		
	No	2.56	(0.55, 12.01)	0.233
Used condom at last sex	Yes	ref	–	–
	No	7.25	(3.06, 17.19)	<0.001
Sex without a condom in last 12 months	Yes	6.20	(0.46, 82.85)	0.168
	No	ref	–	–

Note: PR – prevalence ratio, CI – confidence interval, n/a – insufficient data to calculate effect estimate.

*Respond “Yes” to Form S1 Q17 “Have you exchanged sex for money, goods or services in the last 12 months?” or respond “Yes” to Form S1 Q18 “Have you given money, goods or services in exchange for sex in the last 12 months”.

**Respond “Yes” to Form S1 Q9 “Do you feel stigmatized (ignored, shamed) by your social circle because you participated in this study?”.

### Risk profiles

To assess for risk profiles for recent HIV acquisition, we conducted a latent class analysis. The analysis considered four variables—1) current alcohol consumption, 2) report of ever having sex while drunk or high on drugs, 3) participating in transactional sex in the past 12 months, and 4) not using a condom at last sex—and produced two groups, including a low-risk group and a high-risk group (Fig 3). Study participants were more likely to belong to the low-risk group (69.0%, 95% CI: 58.6, 77.8%) than to the high-risk group (31.0%, 95% CI: 22.2, 41.4%), suggesting the high-risk group was a minority in the study population by self-report. The probability of reporting no condom use at last sex was the only variable for which there was not a significant difference between the high risk (69.9%, 95% CI: 61.1, 78.7%) and low risk (73.9%, 95% CI: 67.4, 80.4%) groups (Fig 3).

**Fig 3:**
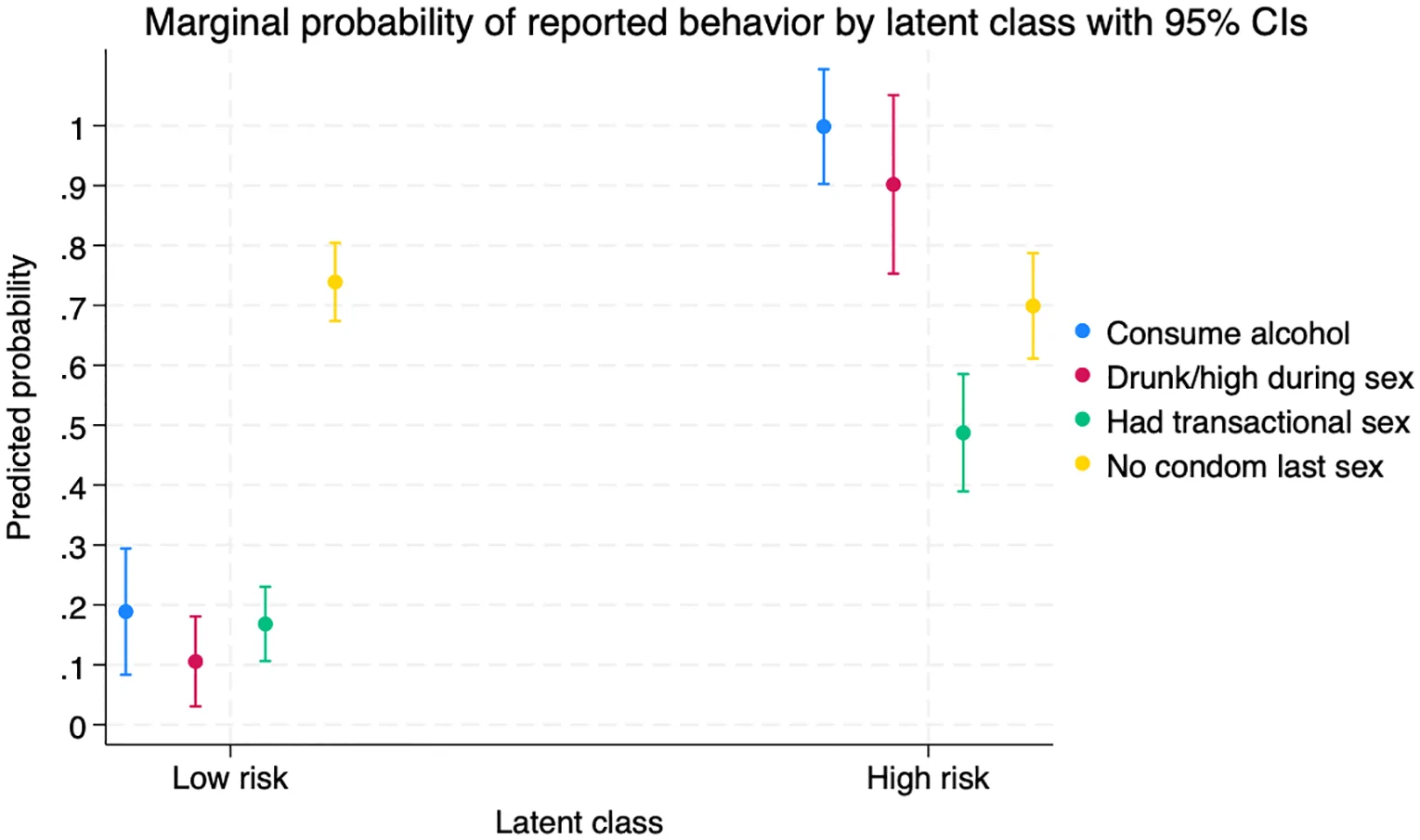
Probability of reporting behavior by latent class (lower risk and higher risk) with 95% confidence intervals (N = 317). Note: CI – confidence interval. *Currently consume alcohol. **Ever had sex while drunk or high. †Transactional sex includes those that responded “Yes” to Q17 or Q18 on Form S1 File.

### Social harm

The social harms survey was completed by 305 (96.2%, n/N = 305/317) participants at a mean of 93.8 days (standard deviation:71.3 days) post-enrolment. Of those 305, 27 (8.9%) individuals reported a total of 29 social harms attributable to recency testing and/or events following study enrollment (Fig 4a). Of those reporting a social harm, four (of the thirty) reported experiencing some sort of social harm at baseline/enrollment and a majority attributed the social harm to the recency testing itself, and specifically the disclosure of the recency test result. The most cited social harm among those indicating ≥1 person reacted badly to their involvement with recency testing was a friend, relative, or other member of their social network saying words that made the participant “feel sad, unhappy, or anxious” (53.8%) followed by someone “ending a relationship with the participant” (25.9%) (Fig 4b).

**Fig 4:**
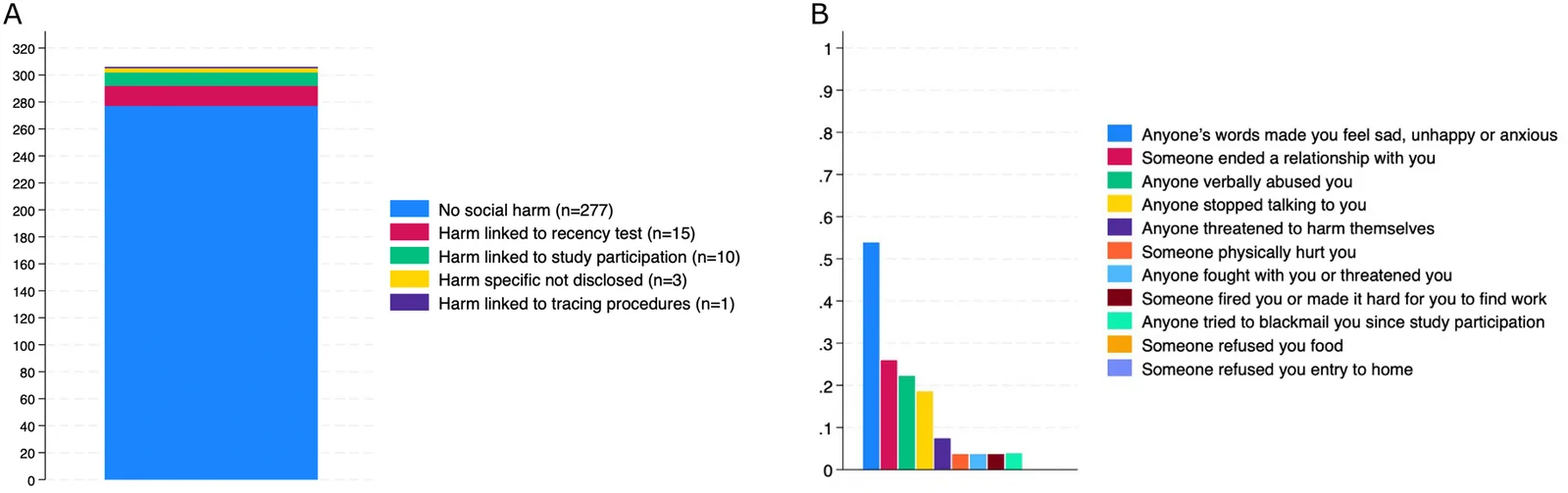
A) Frequency and category of social harm reported during study participation (N = 305); B) Proportion of specific social harms reported by participants and attributed to recent testing or other aspect of study participation (N = 29).

## Discussion

While integrating recency testing into routine index testing was feasible, it did not significantly improve index testing efficiency, as measured by HIV positivity among contacts of recently acquired versus long-term index clients. Approximately 9% of participants reported a social harm following their new HIV diagnosis—potentially related to post-test counseling, linkage to care, assisted partner notification, recency testing, or adjusting to a new diagnosis—with about half attributing it specifically to learning their recency results. Taken together, our results do not support routine individual-level recency testing to inform HIV case finding in Zambia. Any benefits of a more targeted approach might instead be realized by prioritizing services among individuals reporting high-risk behaviors, such as no condom use at last sexual encounter, or by improving contact elicitation and tracing more generally.

We observed similar test positivity among contacts of recently acquired versus long-term index clients. Though relatively few participants were identified as RITA-recent, study staff ensured each completed a second round of contact elicitation. While new contacts can be reported during routine care, this occurs infrequently in practice; as such, contact elicitation likely proceeded with greater fidelity through the study’s added focus on RITA-recent participants. Emerging data suggest that supporting repeated rounds of contact elicitation as standard of care may improve the number of PLHIV newly identified through index testing [28]. Further research should examine whether returning results to clients improves partner elicitation during index testing, or if alternative strategies for multiple contact rounds are more effective, while considering resource constraints.

Our biobehavioral survey identified risk factors associated with recent HIV acquisition, including not using a condom or having a one-time partner at the time of a participant’s last sexual encounter. These factors suggest that condomless sex with a casual partner may be particularly important for identifying persons with recent HIV acquisition, as has been shown in the general HIV epidemiology literature [29–31]. The importance of condom use (or non-use) has been identified previously as a risk factor using machine learning techniques applied to the Zambia Population-based HIV Impact Assessment (ZAMPHIA) [32]. Interestingly, a higher proportion of participants with recently acquired HIV considered themselves to be at “low risk” for HIV prior to their diagnosis, pointing to a mismatch between self-perceived risk and actual risk in settings with ongoing HIV transmission.

We used a latent class analysis to identify profiles of risk for recent HIV acquisition, which revealed that condomless sex in the last year is one of the strongest indicators of recent infection. Our analysis also revealed large differences in probability of reporting alcohol consumption, being drunk or high during sex, and transactional sex between the high-risk and low-risk groups, while the difference in no condom use at last sex was not significantly different across latent classes. This, combined with the strong association between recency and no condom use at last sex, suggests that no condom use at last sex may be the single most relevant and parsimonious behavior to examine when identifying individuals at higher risk for recent HIV acquisition.

Social harm was reported by nearly one in ten participants, largely attributed to recency testing itself, including individual-level disclosure of results. When asked to specify the harm, a majority reported sadness, anxiety, and negative effects on interpersonal relationships. Though physical abuse was rare, more than one in five participants who experienced social harm reported verbal abuse. While data on social harm specific to recency testing are scarce, previous evidence shows partner notification can result in social harm. In sub-Saharan Africa, approximately 1% of the population reported social harm associated with partner notification outside of recency testing, most commonly relationship dissolution, followed by violence and loss of financial support [33–36]. Additional mixed methods research is required to understand the exact timing of their occurrence following recency testing, as well as to what extent these are attributable to recency testing itself versus the cascade of events that occur following a new HIV diagnosis.

We recognize several limitations with this pilot study. First, we have limited power to explore outcomes of interest in study subgroups, such as young women. Accordingly, for all outcomes and subgroups analyzed, we have reported 95% confidence intervals to clarify the limited precision of our effect size estimates. Second, it is possible that there was some social desirability bias in reporting of risk behaviors overall and among those with recently acquired HIV compared to those with long-term infection. Third, we were unable to approach two-thirds of HTS recipients for study participation due to study staffing limitations and the sheer volume of HTS offered at the study sites. This limitation, combined with the fact that our pilot was only done at two urban HIV care facilities in Lusaka, means that our results may not be fully generalizable to all HTS clients or outside similar urban settings in Zambia. Finally, given the heightened risk of potential social harm facing key populations at high risk of HIV who receive HTS, we purposefully did not recruit them for study participation, and so our results may not be applicable to these populations.

## Conclusions

In this pilot observational study, integrating individual-level recency testing, using the national RITA, into routine index testing services did not change the testing positivity of index contacts traced and tested. While this approach was feasible and may have offered a dedicated opportunity for partner re-elicitation, the lack of an observed effect on HIV case finding, coupled with reported incidents of social harm and an absence of known benefit for individual health, argue against the use of individual-level recency testing as a strategy to improve HIV index testing efficiency. Close examination of biobehavioral risk factors associated with recently acquired HIV, such as no condom use at last sex, suggest that careful assessment of individuals’ risk behaviors may provide a safer and more scalable alternative for risk-differentiated case finding in index testing programs and merits further study.

## Supporting information

S1 File REACHZ social harms survey. (PDF)

S2 File REACHZ bio-behavioral survey. (PDF)

S1 Table Index testing results by recent infection testing algorithm result (N = 317). (PDF)

## References

[1] Health ZMo. Zambia population-based HIV impact assessment (ZAMPHIA) 2021: final report. Zambia Statistics Office. 2023.

[2] Lasry A, Medley A, Behel S, Mujawar MI, Cain M, Diekman ST, et al. Scaling Up Testing for Human Immunodeficiency Virus Infection Among Contacts of Index Patients - 20 Countries, 2016-2018. MMWR Morb Mortal Wkly Rep. 2019;68(21):474–7. doi: 10.15585/mmwr.mm6821a2 31145718 PMC6542477

[3] Girault P, Green K, Clement NF, Rahman YAA, Adams B, Wambugu S. Piloting a Social Networks Strategy to Increase HIV Testing and Counseling Among Men Who Have Sex with Men in Greater Accra and Ashanti Region, Ghana. AIDS Behav. 2015;19(11):1990–2000. doi: 10.1007/s10461-015-1069-z 25903507

[4] Golden MR, Gift TL, Brewer DD, Fleming M, Hogben M, St Lawrence JS, et al. Peer referral for HIV case-finding among men who have sex with men. AIDS. 2006;20(15):1961–8. doi: 10.1097/01.aids.0000247118.74208.6a 16988518

[5] Lillie TA, Persaud NE, DiCarlo MC, Gashobotse D, Kamali DR, Cheron M, et al. Reaching the unreached: Performance of an enhanced peer outreach approach to identify new HIV cases among female sex workers and men who have sex with men in HIV programs in West and Central Africa. PLoS One. 2019;14(4):e0213743. doi: 10.1371/journal.pone.0213743 30943205 PMC6447144

[6] WHO. Consolidated guidelines on differentiated HIV testing services. 2024. 39116265

[7] Coldiron ME, Stephenson R, Chomba E, Vwalika C, Karita E, Kayitenkore K, et al. The relationship between alcohol consumption and unprotected sex among known HIV-discordant couples in Rwanda and Zambia. AIDS Behav. 2008;12(4):594–603. doi: 10.1007/s10461-007-9304-x 17705032

[8] Davey JD, Kilembe W, Wall KM, Khu NH, Brill I, Vwalika B. Risky sex and HIV acquisition among HIV serodiscordant couples in Zambia, 2002-2012: What does alcohol have to do with it?. AIDS Behav. 2017;21(7):1892–903. 10.1007/s10461-017-1733-6 PMC5493513 28243934

[9] Surveillance UWWGOGHAaS. When and how to use assays for recent infection to estimate HIV incidence at a population level. 2011. https://apps.who.int/iris/bitstream/handle/10665/44612/9789241501675_eng.pdf

[10] WHO TEAM Global HIV HaSTIP. Using recency assays for HIV surveillance: 2022 technical guidance. WHO. 2022. https://www.who.int/publications/i/item/9789240064379

[11] Cohen MS, Shaw GM, McMichael AJ, Haynes BF. Acute HIV-1 Infection. N Engl J Med. 2011;364(20):1943–54. 10.1056/NEJMra1011874 PMC3771113 21591946

[12] Powers KA, Ghani AC, Miller WC, Hoffman IF, Pettifor AE, Kamanga G, et al. The role of acute and early HIV infection in the spread of HIV and implications for transmission prevention strategies in Lilongwe, Malawi: a modelling study. Lancet. 2011;378(9787):256–68. doi: 10.1016/S0140-6736(11)60842-8 21684591 PMC3274419

[13] Wawer MJ, Gray RH, Sewankambo NK, Serwadda D, Li X, Laeyendecker O, et al. Rates of HIV-1 transmission per coital act, by stage of HIV-1 infection, in Rakai, Uganda. J Infect Dis. 2005;191(9):1403–9. doi: 10.1086/429411 15809897

[14] Rice BD, de Wit M, Welty S, Risher K, Cowan FM, Murphy G, et al. Can HIV recent infection surveillance help us better understand where primary prevention efforts should be targeted? Results of three pilots integrating a recent infection testing algorithm into routine programme activities in Kenya and Zimbabwe. J Int AIDS Soc. 2020;23 Suppl 3(Suppl 3):e25513. doi: 10.1002/jia2.25513 32602625 PMC7325505

[15] Godin A, Eaton JW, Giguère K, Marsh K, Johnson LF, Jahn A, et al. Inferring population HIV incidence trends from surveillance data of recent HIV infection among HIV testing clients. AIDS. 2021;35(14):2383–8. doi: 10.1097/QAD.0000000000003021 34261098 PMC8631145

[16] Zhu Q, Wang Y, Liu J, Duan X, Chen M, Yang J, et al. Identifying major drivers of incident HIV infection using recent infection testing algorithms (RITAs) to precisely inform targeted prevention. Int J Infect Dis. 2020;101:131–7. doi: 10.1016/j.ijid.2020.09.1421 32987184

[17] Columbia I. HIV recency testing can identify new infections earlier, ICAP and Rwanda Biomedical Center evaluation finds. 2024. https://icap.columbia.edu/news-events/hiv-recency-testing-can-identify-new-infections-earlier-icap-and-rwanda-biomedical-center-evaluation-finds/

[18] Nikolopoulos GK, Pavlitina E, Muth SQ, Schneider J, Psichogiou M, Williams LD, et al. A network intervention that locates and intervenes with recently HIV-infected persons: The Transmission Reduction Intervention Project (TRIP). Sci Rep. 2016;6:38100. doi: 10.1038/srep38100 27917890 PMC5137009

[19] (TRACE) TwRAtCtE. Tracking with recency assays to control the epidemic. 2023. https://trace-recency.org

[20] HIV/AIDS UJUNPo. Ending the epidemic: 2025 AIDS targets. 2019.

[21] PEPFAR. PEPFAR 2022 Country and Regional Operational plan (COP/ROP) guidance for all PEPFAR-supported countries. 2022.

[22] Health ZMo. Zambia Consolidated Guidelines for Treatment and Prevention of HIV Infection. 2022.

[23] Health ZMo. Zambia Consolidated Guidelines for Treatment and Prevention of HIV Infection. 2020.

[24] Health ZMo. Zambia Consolidated Guidelines for the Treatment and Prevention of HIV Infection. 2022.

[25] Yufenyuy EL, Detorio M, Dobbs T, Patel HK, Jackson K, Vedapuri S, et al. Performance evaluation of the Asante Rapid Recency Assay for verification of HIV diagnosis and detection of recent HIV-1 infections: Implications for epidemic control. PLOS Glob Public Health. 2022;2(5):e0000316. doi: 10.1371/journal.pgph.0000316 36962217 PMC10021762

[26] Browne FA, Wechsberg WM. The intersecting risks of substance use and HIV risk among substance-using South African men and women. Curr Opin Psychiatry. 2010;23(3):205–9. doi: 10.1097/YCO.0b013e32833864eb 20308902 PMC3784346

[27] Vinikoor MJ, Sikazwe I, Sharma A, Kanguya T, Chipungu J, Murray LK, et al. Intersection of alcohol use, HIV infection, and the HIV care continuum in Zambia: nationally representative survey. AIDS Care. 2023;35(10):1555–62. doi: 10.1080/09540121.2022.2092589 35761776 PMC9792627

[28] Musee P, Onyango CA, Yemaneberhan A, Ethridge M. Intensified HIV Case Finding through Index Case Testing in Kenya: A Model of Success. 2020.

[29] Hargreaves JR, Morison LA, Kim JC, Busza J, Phetla G, Porter JDH, et al. Characteristics of sexual partnerships, not just of individuals, are associated with condom use and recent HIV infection in rural South Africa. AIDS Care. 2009;21(8):1058–70. doi: 10.1080/09540120802657480 20024763

[30] Sebogodi MR, Huma M, Mokgatle MM, Mathebula MP, Nchabeleng M. Casual Sex, Sex Work and Unprotected Sex among Men Who Have Sex with Men in the Human Papilloma Virus (HPV) Clinical Trial —North-West Region of Tshwane, South Africa. WJA. 2019;09(04):167–82. doi: 10.4236/wja.2019.94013

[31] Kalichman SC, Ntseane D, Nthomang K, Segwabe M, Phorano O, Simbayi LC. Recent multiple sexual partners and HIV transmission risks among people living with HIV/AIDS in Botswana. Sex Transm Infect. 2007;83(5):371–5. doi: 10.1136/sti.2006.023630 17475684 PMC2659030

[32] Chingombe I, Musuka G, Mbunge E, Chemhaka G, Cuadros DF, Murewanhema G, et al. Predicting HIV Status Using Machine Learning Techniques and Bio-Behavioural Data from the Zimbabwe Population-Based HIV Impact Assessment (ZIMPHIA15-16). Lecture Notes in Networks and Systems. Springer International Publishing. 2022. p. 247–58. doi: 10.1007/978-3-031-09076-9_24

[33] Greensides D, Bishop K, Manfredini L, Wong V. Do No Harm: A Review of Social Harms Associated with HIV Partner Notification. Glob Health Sci Pract. 2023;11(6):e2300189. doi: 10.9745/GHSP-D-23-00189 38135515 PMC10749648

[34] Mutale W, Freeborn K, Graybill LA, Lusaka MM, Mollan KR, Mweemba O, et al. Addition of HIV self-test kits to partner notification services to increase HIV testing of male partners of pregnant women in Zambia: two parallel randomised trials. Lancet Glob Health. 2021;9(12):e1719–29. doi: 10.1016/S2214-109X(21)00393-4 34735862 PMC8644317

[35] Plotkin M, Kahabuka C, Christensen A, Ochola D, Betron M, Njozi M, et al. Outcomes and Experiences of Men and Women with Partner Notification for HIV Testing in Tanzania: Results from a Mixed Method Study. AIDS Behav. 2018;22(1):102–16. doi: 10.1007/s10461-017-1936-x 29090397 PMC5758678

[36] Rosenberg NE, Mtande TK, Saidi F, Stanley C, Jere E, Paile L, et al. Recruiting male partners for couple HIV testing and counselling in Malawi’s option B+ programme: an unblinded randomised controlled trial. Lancet HIV. 2015;2(11):e483-91. doi: 10.1016/S2352-3018(15)00182-4 26520928 PMC4656790

