# Supplementary material for: Results of integrating recency testing into routine index testing services in Lusaka, Zambia: A mixed effects regression analysis
Source: PLoS One. 2026 Oct 30;21(10):e0361598. doi: 10.1371/journal.pone.0361598 (PMC13722200; doi:10.1371/journal.pone.0361598)
Supplement: Supplementary file 1 [file pone.0361598.s001.pdf]

Date of Visit: ☐☐/☐☐☐/☐☐☐☐ Date of Birth: ☐☐/☐☐☐/☐☐☐☐

dd/MM/yyyy *dd/MM/yyyy*

Participant Sex : M ☐ F ☐ Prefer not to answer ☐ Site name ________________________

PTID ☐☐☐☐

REACHZ Bio-behavioral Survey

Instructions to Interviewer:

Ask all questions of all participants, except if the question specifically notes to skip
Read the question exactly as printed
Politely wait for answer

Remind participants that they do not have to answer any question if they do not want to

Do not leave a question unanswered.

When code not specifically listed, use these codes if necessary: 77 = Refused, 88 = Don't know, 99= Not applicable

Opening script: "*I am going to ask you questions about your life, experiences, behavior, sexual history and any clinical symptoms. We are trying to understand someone's risk factors for getting infected with HIV. Please try your best to answer every question truthfully, to the best of your knowledge. You do not have to answer any questions that you do not want to anwer.***"**

Q1. How old are you? .... (age in years) .... ☐☐

Q2. What is your biological sex? .... ☐☐

01 Male
02 Female
03 Intersex

Q3. What do you consider your gender? .... ☐☐

01 Male
02 Female
03 Transgender
04 Other

Q4. Do you have a mobile phone? .... ☐☐

01 Yes
02 No

Q5. Do you use the internet? .... ☐☐

01 Yes
02 No

Q6. What is the highest level of school/ college you have completed?

.... ☐☐

01 Lower primary school (Grade 1-4)
02 Upper primary school (Grade 5-7)
03 Junior secondary (Grade 8-9)
04 Senior secondary (Grade10-12)
05 College/university
99 Never been to school

Q7. What is the main occupation or activity through which you earn money? (**Note to RA**: please do not read out this list, pick the answer closest to the one provided)

.... ☐☐

01 No income/ not employed
02 Full time student
03 Self employed
04 Unpaid family worker
05 House maid/ home maker
06 In wage employment/ formal employment
07 Not working/ looking for work
08 Not working/ not looking for work
09 Retired

10 Sex work
11 Drug dealing
12 Other
13 Would rather not say

Q8. What is your marital status? .... ☐☐

01 Single
02 Married
03 Divorced/ Separated
04 Widowed

Q9. Who do you currently live with? (Note to RA: Do not read answers, record all that are mentioned)

05 I live alone
06 Parents
07 Wife/ Husband
08 Sexual Partner
09 Siblings
10 Children
11 Other family / relatives
12 Friend or roommate (not sexual partner)
13 Other

Q10. Would you describe yourself as a member of a key population (female sex worker, men who have sex with men, transgender person, person who uses injection drugs)?

.... ☐☐

01 Yes
02 No
03 Would rather not say

Q11. Have you injected or someone injected you with drugs that were not prescribed by a doctor? .... ☐☐ (If answer is no, skip to 14)

01 Yes
02 No

88 Do not know

Q12. Have you ever shared a needle with someone when injecting drugs?

.... ☐☐

01 Yes
02 No
88 Do not know

Q13. Do you know where to access clean needles in your catchment area?

.... ☐☐

01 Yes
02 No

Q14. Do you consume alcohol? .... ☐☐ (if No, skip to 16)

89 Yes
90 No

Q15. How much alcohol do you drink in a week? .... ☐☐

01 1-2 drinks
02 3-5 drinks
03 5-7 drinks
04 More than 7 drinks

Q16. Have you ever had sex while drunk or high on drugs? .... ☐☐

01 Yes
02 No

Q17. Have you exchanged sex for money, goods or services in the last 12 months?

.... ☐☐

01 Yes
02 No

Q18. Have you given money, goods or services in exchange for sex in the last 12 months?

.... ☐☐

01 Yes
02 No

Q19. Have you had unprotected sex in the last 12 months? (unprotected = without a condom)

.... ☐☐

01 Yes
02 No

Q20. In the past 12 months, how many sexual partners have you had?

.... ☐☐

01 None
02 1-3
03 3-6
04 6+

Q21. Have you had receptive anal sex in the last 12 months (where someone inserts their penis into your anus)? .... ☐☐

01 Yes
02 No
03 Would rather not say

Q22. Have you had insertive anal sex in the last 12 months (where you put your penis into someone's anus)? .... ☐☐

01 Yes
02 No
03 Would rather not say

Q23. Where or how do you normally meet sexual partners? (**Note to RA**: Do not read the answers, just check all that apply)

01 Brothel
02 Bar/ Disco
03 Café/ Restaurant
04 Street/ park
05 Through friends
06 Internet (facebook, whatsapp, chat)
07 Motel
08 School
09 Party/ social gathering
10 Through an intermediary (pimp, bartender, taxi driver, security guard)
11 Service station
12 Truck stop, rail station, public transport
13 Border crossing
14 other

Q24. What type of partner was your last partner? .... ☐☐

01 Regular
02 Casual
03 One time only
04 Sex work client

Q25. Thinking about your last sexual partner, what did you know or believe their HIV status to be? .... ☐☐

01 Negative
02 Positive
88 Do not know

Q26. Do you know where to access condoms in your catchment area? .... ☐☐

01 Yes
02 No

Q27. Did you use a condom with this partner? (if Yes, skip to 29) .... ☐☐

01 Yes
02 No
88 Do not know

Q28. If you did not use a condom, why not? (**Note to RA**: Please do not read the answers, pick the one that matches the description)

01 No condom available
02 Partner refused
03 Condoms reduce pleasure
04 Used other contraceptives
05 Not worried about getting HIV/ other STIs
06 Condoms break/ don't work
07 Wanted to get pregnant
08 Got paid more to have sex without a condom
09 Forced to have sex without a condom
10 Other (specify) ____________________

Q29. If a condom was used, why was it used? .... ☐☐

01 To prevent HIV/ STIs
02 Do not trust partner
03 Messages advising the use of condoms
04 To prevent pregnancy
05 Other
88 Don't know

Q30. Was your last sexual partner a person who injects drugs? .... ☐☐

01 Yes
02 No
88 Do not know

Q31. What do you think is the risk of you getting HIV? .... ☐☐

01 Low
02 Medium
03 High
88 Don't know

Q32. Have you ever been tested for HIV in the past and received results? ..... ☐☐

01 Yes
02 No
88 Don't know

Q33. Have you ever heard about PrEP? (PrEP is the antiretroviral medicine taken every day to prevent one from getting HIV) .... ☐☐ (if No, skip to 36)

01 Yes
02 No

Q34. Have you ever been recommended to begin PrEP? .... ☐☐

01 Yes
02 No

Q35. Have you ever taken PrEP? .... ☐☐

01 Yes
02 No

Q36. Have you ever taken PEP? (PEP is antiretroviral medicine to prevent HIV infection if you think you have had a high-risk exposure) .... ☐☐

01 Yes
02 No
88 Do not know

Q37. Have you had a genital sore (sore near the penis or vagina) in the last 12 months? .... ☐☐

01 Yes
02 No
88 Do not know

Q38. Have you had an abnormal discharge from your penis or vagina in the last 12 months? .... ☐☐

01 Yes
02 No
88 Do not know

39. Have you been examined for an STI (other than HIV) in the last 12 months? .... ☐☐

01 Yes
02 No
88 Do not know

Q40. Have you had symptoms of possible TB (cough, fever, night sweats, weight loss)?

.... ☐☐

01 Yes
02 No
88 Do not know
