# Supplementary material for: Results of integrating recency testing into routine index testing services in Lusaka, Zambia: A mixed effects regression analysis
Source: PLoS One. 2026 Oct 30;21(10):e0361598. doi: 10.1371/journal.pone.0361598 (PMC13722200; doi:10.1371/journal.pone.0361598)
Supplement: Supplementary file 2 [file pone.0361598.s002.pdf]

Date of Visit (*DD/MMM/YYYY*): □□/□□□/□□□□ PTID: □□□-□□□□

REACHZ Social harms survey
Instructions to Interviewer:

Ask all questions of all participants, except if the question specifically notes to skip. Read the question exactly as printed. Politely wait for answer

Remind participants that they do not have to answer any question if they do not want to.

Do not leave a question unanswered.

Opening script: "*I am going to ask you questions about problems that you may have experienced in the last several months. We are trying to understand if any of these problems occurred because of your enrolment in the study and of the steps in recency testing, i.e. undergoing a recency test, getting your recency test result, or healthcare workers contacting and testing your partner or people from your social network. Please try your best to answer every question truthfully, to the best of your knowledge. You do not have to answer any questions that you do not want to anwer.***"**

Q1. Please think of people who know about your participation in this study or about your taking a recency test. Of these people, who are the top 5 people who reacted badly to your study participation or taking a recency test?

No.	Name of Person	Relationship to you	Did this person react badly to your study participation?	Did this person react badly to your recency test result?	Did this person react badly to being contacted by HCW?
1					
2					
3					
4					
5					

Q2. Thinking about the people you said found out about your participation in the study, how did the person (or people) involved find out that you either participated in the study or were recently infected?

Please mention one reason for each of the people you mentioned above.

01 Participant told them

02 Research study staff told them
03 Healthcare worker told them
04 Found study material
05 Found medication
06 Saw participant at the clinic
07 Was told by someone else in the community that the participant had disclosed to
08 Was told by someone else in the community that the participant HAD NOT disclosed to
09 Don't know
10 Other (please specify) ___________

No.	Reason Code
1	
2	
3	
4	
5	

Q3. Now, thinking about these people and how they may have hurt you, please answer the following questions.

Interviewer: For all Qs answered as Yes, please ask who among the 5 did this. Then ask if anyone else hurt them in any way. Please note relationship of the person (s) who did this.

Since you took part in this study:

Q3.	Question/Type of harm	Response	1	2	3	4	5	Other (multiple responses allowed)
A	Has someone ended a relationship with you?	☐ Yes<br>☐ No → SKIP TO B						
B	Has someone physically hurt you (hurt = pushed, shoved, beaten, hurt with weapon, slapped)?	☐ Yes<br>☐ No → SKIP TO C						
C	Has anyone fought with you or threatened you?	☐ Yes<br>☐ No → SKIP TO D						
D	Has anyone threatened to harm themselves (e.g., attempt suicide)?	☐ Yes<br>☐ No → SKIP TO E						

E	Has anyone verbally abused you (e.g., shout or curse)?	☐ Yes<br>☐ No → SKIP TO F						
F	Has anyone stopped talking to you?	☐ Yes<br>☐ No → SKIP TO G						
G	Has anyone refused you food?	☐ Yes<br>☐ No → SKIP TO H						
H	Has anyone refused you access to entry to your house?	☐ Yes<br>☐ No → SKIP TO I						
I	Has someone fired you or made it hard for you to find work?	☐ Yes<br>☐ No → SKIP TO J						
J	Has anyone tried to blackmail you since you participated in this study? (*Blackmail means that someone is asking for money or some other benefit from you in return for not revealing that you either participated in this study or that you were recently infected)*	☐ Yes<br>☐ No → SKIP TO K						
K	Has anyone's words made you feel sad, unhappy or anxious?	☐ Yes<br>☐ No → SKIP TO Q4						

Q4. Thinking about all the situations you have just described, please identify the one situation that has caused you the most distress.
(NOTE TO RA: Please let the participant identify an example that has affected them most. Please do not read this list to them. Please pick from the list below which answer most closely represents the participant's answer) ....☐☐

01 Ending the relationship
02 Physical abuse
03 Partner fighting with you
04 Partner threatening to hurt themselves
05 Verbal abuse from social circle

06 Social circle member ignoring you
07 Denied access to food and housing
08 Someone’s words/ actions caused you to feel unhappy or depressed
09 Loss of job or financial stress
10 Blackmail

Q5. How would you describe the impact of this situation on your day-to-day life?

...............................☐☐

01 Has had no impact on my life
02 Has caused a little impact on my life
03 Has had medium impact on my life
04 Has caused huge impact on my day-to-day life

Q6. Do you think this situation can be resolved or is it permanent?

...............................☐☐

01 Has already been resolved
02 Can be resolved
03 Will not be resolved
04 Cannot say

Q7. Thinking again about this situation, why do you think the person who did this behaved the way they did towards you?

(NOTE TO RA: Do not read out the choices below, have the participant respond and then select ALL reasons that apply. If additional reasons are given. Please provide below.)

01 They thought I was cheating on them ☐

02 They thought I had brought shame on the family/ community ☐

03 They were worried that I would (or already had) infected them ☐

04 They were angry that I had engaged with healthcare workers ☐

05 They don’t approve of my (sexual) lifestyle (e.g. MSM, sex worker, transgender, etc) ☐

06 They thought that I had been promiscuous ☐

07 They thought I was lying ☐

Please specify any other reason:

Thinking now about how your participation in the study has made you feel, please answer the next few questions.

Think about how you feel RIGHT NOW or how you have felt in the last three months.

Q8. Do you feel fearful or scared about harms that may come to you because you joined this study? .... ☐☐

01 Yes
02 No

Q9. Do you feel stigmatized (ignored, shamed) by your social circle because you participated in this study? .... ☐☐

01 Yes
02 No

Q10. Have you thought about harming yourself (e.g. attempting suicide, cutting yourself, deliberately causing an injury) since you participated in study?

.... ☐☐

01 Yes
02 No

(*NOTE to RA: If a participant answers YES to this question, please activate Handling social harms SOP and make a plan to accompany the participant to speak with a health worker).*

Q11. Over the last three months (since you participated in the study), have you felt sad, depressed, or had no interest in doing things? .... ☐☐

01 Yes
02 No

Thank the participant for their time.

Closing script: "*Thank you for your time and for sharing your experiences with me today. If you have any further questions, please feel free to talk to me or the PI of the study"*

Completed by _____________ Checked by___________ Entered by _____________

Completed on_____________ Checked on___________ Entered on_____________
