# Supplementary material for: Results of integrating recency testing into routine index testing services in Lusaka, Zambia: A mixed effects regression analysis
Source: PLoS One. 2026 Oct 30;21(10):e0361598. doi: 10.1371/journal.pone.0361598 (PMC13722200; doi:10.1371/journal.pone.0361598)
Supplement: Supplementary file 3 [file pone.0361598.s003.pdf]

Supplemental material

Results of integrating recency testing into routine index testing services in Lusaka, Zambia: A mixed effects regression analysis

Jake M. Pry[1,2*], Shilpa S. Iyer[1,3*], Chilambwe Mwila[1], Christiana Frimpong[1], Misinzo Moono[1], Musunge Mulabe[1], Mirriam Nanyangwe[1], Herbert Kapesa[1], Lumbani Phiri[1], Patricia Maritim[1], Melissa M. Arons[4], Tiffiany M. Aholou[4], Carolyn Bolton Moore[1,5], Theodora Savory[1], Michael E. Herce[1,6]

1 Centre for Infectious Disease Research in Zambia (CIDRZ), Lusaka, Zambia
2 School of Medicine, University of California, Davis, California, USA
3 Global Vaccine Preventable Diseases Laboratory Networks, World Health Organization, Geneva, Switzerland
4 Division of Global HIV and TB, Global Health Center, U.S. Centers for Disease Control and Prevention, Atlanta, USA
5 School of Medicine, University of Alabama, Birmingham, Alabama, USA
6 Institute for Global Health & Infectious Diseases, University of North Carolina, Chapel Hill, North Carolina, USA

*Co-first authors/ contributed equally to this work.

S1 Table. Index testing results by recent infection testing algorithm result (N=317).

**Contact status and test result**	**RITA Long-term (n=297)**	**RITA Recent (n=20)**		
	**Initial elicitation**	**Initial elicitation**	**Re-elicitation**	**Total elicitation**
	n (%)	*n (%)*	*n (%)*	*n (%)*
Reported contacts	479	39	14	53
Elicitation [ratio]	[1.61]	[1.95]	[0.7]	[2.65]
Known positive	165 (34.4)	16 (41.0)	3 (21.4)	19 (35.8)
Tracing failed	74 (15.4)	3 (7.7)	3 (21.4)	6 (11.3)
Tested	240 (50.1)	20 (51.3)	8 (57.1)	28 (52.8)
Negative	173 (72.1)	14 (70.0)	6 (75.0)	20 (71.4)
Positive	67 (27.9)	6 (30.0)	2 (25.0)	8 (28.6)

Note: RITA – recent infection testing algorithm
