# Supplementary material for: Results of integrating recency testing into routine index testing services in Lusaka, Zambia: A mixed effects regression analysis
Source: PLoS One. 2026 Oct 30;21(10):e0361598. doi: 10.1371/journal.pone.0361598 (PMC13722200; doi:10.1371/journal.pone.0361598)
Supplement: Peer review history

# Peer review: Decision Letter 0

**DOI:** 10.1371/journal.pone.0361598.r001

**Authors:** Sahjid Mukhida

21 Aug 2026

Dear Dr. Pry,

Thank you for submitting your manuscript to PLOS One. After careful consideration, we feel that it has merit but does not fully meet PLOS One’s publication criteria as it currently stands. Therefore, we invite you to submit a revised version of the manuscript that addresses the points raised during the review process.

Please submit your revised manuscript by Oct 05 2026 11:59PM. If you will need more time than this to complete your revisions, please reply to this message or contact the journal office at plosone@plos.org. When you’re ready to submit your revision, log on to https://www.editorialmanager.com/pone/ and select the 'Submissions Needing Revision' folder to locate your manuscript file.

- A letter that responds to each point raised by the academic editor and reviewer(s). You should upload this letter as a separate file labeled 'Response to Reviewers'.
- A marked-up copy of your manuscript that highlights changes made to the original version. You should upload this as a separate file labeled 'Revised Manuscript with Track Changes'.
- An unmarked version of your revised paper without tracked changes. You should upload this as a separate file labeled 'Manuscript'.

If you would like to make changes to your financial disclosure, please include your updated statement in your cover letter. Guidelines for resubmitting your figure files are available below the reviewer comments at the end of this letter.

If applicable, we recommend that you deposit your laboratory protocols in protocols.io to enhance the reproducibility of your results. Protocols.io assigns your protocol its own identifier (DOI) so that it can be cited independently in the future. For instructions see: https://journals.plos.org/plosone/s/submission-guidelines#loc-laboratory-protocols. Additionally, PLOS One offers an option for publishing peer-reviewed Lab Protocol articles, which describe protocols hosted on protocols.io. Read more information on sharing protocols at https://plos.org/protocols?utm_medium=editorial-email&utm_source=authorletters&utm_campaign=protocols.

As the corresponding author, your ORCID iD is verified in the submission system and will appear in the published article. PLOS supports the use of ORCID, and we encourage all coauthors to register for an ORCID iD and use it as well. Please encourage your coauthors to verify their ORCID iD within the submission system before final acceptance, as unverified ORCID iDs will not appear in the published article. *Only*  the individual author can complete the verification step; PLOS staff *cannot*  verify ORCID iDs on behalf of authors.

We look forward to receiving your revised manuscript.

Kind regards,

Sahjid Mukhida

Academic Editor

PLOS One

Journal Requirements:

When submitting your revision, we need you to address these additional requirements.

1.Please ensure that your manuscript meets PLOS ONE's style requirements, including those for file naming. The PLOS ONE style templates can be found at

https://journals.plos.org/plosone/s/file?id=wjVg/PLOSOne_formatting_sample_main_body.pdf and

https://journals.plos.org/plosone/s/file?id=ba62/PLOSOne_formatting_sample_title_authors_affiliations.pdf

2. We note that the grant information you provided in the ‘Funding Information’ and ‘Financial Disclosure’ sections do not match.

When you resubmit, please ensure that you provide the correct grant numbers for the awards you received for your study in the ‘Funding Information’ section.

3. Thank you for stating the following financial disclosure:

“Funding for this work was provided through the Centers for Disease Control and Prevention (CDC) and the President’s Emergency Plan For AIDS Relief (PEPFAR) through grant number: NU2GGH001920.”

Please state what role the funders took in the study.  If the funders had no role, please state: "The funders had no role in study design, data collection and analysis, decision to publish, or preparation of the manuscript."

If this statement is not correct you must amend it as needed.

Please include this amended Role of Funder statement in your cover letter; we will change the online submission form on your behalf.

4. Thank you for stating the following in your Competing Interests section:

“None declared.”

Please complete your Competing Interests on the online submission form to state any Competing Interests. If you have no competing interests, please state "The authors have declared that no competing interests exist.", as detailed online in our guide for authors at http://journals.plos.org/plosone/s/submit-now

This information should be included in your cover letter; we will change the online submission form on your behalf.

5. Thank you for stating the following in the Acknowledgments Section of your manuscript:

“We would like to thank the study participants who volunteered for the study. We also thank the Zambian Ministry of Health and the Lusaka Provincial Health Officer for their leadership on all facets of the national HIV programme. We would also like to thank the healthcare workers and CIDRZ research staff who made this study possible.”

We note that you have provided funding information that is not currently declared in your Funding Statement. However, funding information should not appear in the Acknowledgments section or other areas of your manuscript. We will only publish funding information present in the Funding Statement section of the online submission form.

Please remove any funding-related text from the manuscript and let us know how you would like to update your Funding Statement. Currently, your Funding Statement reads as follows:

“Funding for this work was provided through the Centers for Disease Control and Prevention (CDC) and the President’s Emergency Plan For AIDS Relief (PEPFAR) through grant number: NU2GGH001920.”

Please include your amended statements within your cover letter; we will change the online submission form on your behalf.

6. Please note that your Data Availability Statement is currently missing the repository name and/or the DOI/accession number of each dataset OR a direct link to access each database. If your manuscript is accepted for publication, you will be asked to provide these details on a very short timeline. We therefore suggest that you provide this information now, though we will not hold up the peer review process if you are unable.

7. Please include captions for your Supporting Information files at the end of your manuscript, and update any in-text citations to match accordingly. Please see our Supporting Information guidelines for more information: http://journals.plos.org/plosone/s/supporting-information.

8. If the reviewer comments include a recommendation to cite specific previously published works, please review and evaluate these publications to determine whether they are relevant and should be cited. There is no requirement to cite these works unless the editor has indicated otherwise.

Additional Editor Comments:

To author team

Thanks for asking me about reviewer name suggestion.

Currently we are going with the available review reports.

Kindly submit atleast 2-3 potential reviewers' name which can review for the journal. Kindly keep in mind, that they should be from other institute/region and not be your recent past co-authors. It is not necessary that we will send your manuscript to them for review.

Thanks and regards

Best wishes

[Note: HTML markup is below. Please do not edit.]

Reviewer's Responses to Questions

**Comments to the Author**

1. Is the manuscript technically sound, and do the data support the conclusions?

Reviewer #1: Partly

**********

2. Has the statistical analysis been performed appropriately and rigorously?

Reviewer #1: I Don't Know

**********

3. Have the authors made all data underlying the findings in their manuscript fully available?

The PLOS Data policy

Reviewer #1: Yes

**********

4. Is the manuscript presented in an intelligible fashion and written in standard English?

Reviewer #1: No

**********

Reviewer #1: The manuscript has valuable programmatic findings, particularly the feasibility of integrating RITA into routine services and the potential importance of behavioral risk assessment. However, the small RITA-recent sample, observational comparison, differential contact elicitation, limited facility number, and uncertainty surrounding attribution of social harms should be addressed before the conclusions can be considered sufficiently robust.

1) The study addresses an important and timely question regarding the integration of HIV recency testing into index testing services. However, the authors should clarify more explicitly what incremental benefit individual-level recency testing is expected to provide over existing risk-based index testing approaches.

2) 20 of 317 participants (6.3%) were classified as RITA-recent, resulting in very small numbers for several key analyses. The authors should emphasize the limited statistical power and interpret the estimates, particularly the large prevalence ratios and wide confidence intervals, with appropriate caution.

3) The observational design does not permit a definitive conclusion that recency testing “did not improve” index-testing efficiency. Since RITA-recent participants received additional partner elicitation whereas long-term participants did not, differences may reflect selection and intervention effects rather than recency status alone.

4) The comparison of HIV positivity among contacts should account more explicitly for differences in the number of contacts elicited and the proportion successfully tested between RITA-recent and long-term groups. The markedly smaller number of contacts in the recent group makes direct comparison potentially unstable.

5) The manuscript describes a mixed-effects Poisson regression with facility-level random effects, but only two study facilities were included. The authors should justify the use and reliability of random-effects estimation with only two clusters or clarify whether facility clustering was handled differently in the relevant analyses.

6) The association between condomless sex and recent HIV acquisition is striking but should not be interpreted as establishing a causal relationship. The authors should also discuss the possibility of recall and social-desirability bias in self-reported sexual behaviors, particularly given the sensitive nature of the questions.

7) The latent class analysis identifies two behavioral profiles, but the relatively small number of participants classified as RITA-recent limits the ability to establish a robust relationship between latent classes and recent infection. Additional justification for the selected number of classes and model fit would strengthen this analysis.

8) The finding that 8.9% of participants reported social harm is important, but attribution specifically to recency testing remains uncertain because participants were newly diagnosed with HIV and also underwent partner notification and other post-diagnosis procedures. The Discussion appropriately acknowledges this issue, but the Abstract and Conclusion should avoid implying that these harms were necessarily caused by recency testing itself.

9) The manuscript should discuss more explicitly that a RITA-recent classification represents an epidemiological estimate of recent infection rather than definitive determination of when or from whom HIV was acquired. This distinction is particularly important when individual-level results are returned and may influence perceptions regarding sexual partners or transmission.

10) The exclusion of populations considered at heightened risk of social harm, together with recruitment from only two urban facilities in Lusaka, substantially limits generalizability. The authors acknowledge this limitation, but it should be emphasized when proposing risk-based behavioral screening as an alternative strategy.

**********

what does this mean?). If published, this will include your full peer review and any attached files.

If you choose “no”, your identity will remain anonymous but your review may still be made public.

**Do you want your identity to be public for this peer review?**  For information about this choice, including consent withdrawal, please see our Privacy Policy

Reviewer #1: **Yes:**  Dr Nikunja Kumar Das

**********

[NOTE: If reviewer comments were submitted as an attachment file, they will be attached to this email and accessible via the submission site. Please log into your account, locate the manuscript record, and check for the action link "View Attachments". If this link does not appear, there are no attachment files.]

To ensure your figures meet our technical requirements, please review our figure guidelines: https://journals.plos.org/plosone/s/figures

You may also use PLOS’s free figure tool, NAAS, to help you prepare publication quality figures: https://journals.plos.org/plosone/s/figures#loc-tools-for-figure-preparation.

NAAS will assess whether your figures meet our technical requirements by comparing each figure against our figure specifications.

# Author comment: Author response to Decision Letter 1

**DOI:** 10.1371/journal.pone.0361598.r002

3 Sep 2026

Dear Dr. Mukhida,

On behalf of my colleagues in Zambia and the United States, I wanted to thank you for the opportunity to respond to the helpful comments you provided as the handling editor. We have reviewed all comments carefully and have revised the manuscript accordingly.

We have enclosed two versions of the revised manuscript, one with changes tracked under “Revised Manuscript with Track Changes” and a second with changes accepted as a “Clean Manuscript”. Please find here our point-by-point responses in italicized blue font to the comments taken from your 22 August 2026 email. Please note that for ease of reference, any line references in the comments below refer to the new “clean” version of the revised manuscript.

****Begin itemized review response

Academic Editor

Journal Requirements:

When submitting your revision, we need you to address these additional requirements.

1.Please ensure that your manuscript meets PLOS ONE's style requirements, including those for file naming. The PLOS ONE style templates can be found at https://journals.plos.org/plosone/s/file?id=wjVg/PLOSOne_formatting_sample_main_body.pdf and https://journals.plos.org/plosone/s/file?id=ba62/PLOSOne_formatting_sample_title_authors_affiliations.pdf

Response: We have reviewed and amended accordingly.

2. We note that the grant information you provided in the ‘Funding Information’ and ‘Financial Disclosure’ sections do not match. When you resubmit, please ensure that you provide the correct grant numbers for the awards you received for your study in the ‘Funding Information’ section.

Response: The only difference identified with the guidance provided is that we had added CDC as the distributor of the PEPFAR funding – we have removed that. If there was something substantial that needs to be changed please provide additional guidance.

3. Thank you for stating the following financial disclosure:

“Funding for this work was provided through the Centers for Disease Control and Prevention (CDC) and the President’s Emergency Plan For AIDS Relief (PEPFAR) through grant number: NU2GGH001920.”

Please state what role the funders took in the study. If the funders had no role, please state: "The funders had no role in study design, data collection and analysis, decision to publish, or preparation of the manuscript."

If this statement is not correct you must amend it as needed.

Please include this amended Role of Funder statement in your cover letter; we will change the online submission form on your behalf.

Response: We have amended the statement slightly and edited the funding statement as follows “The funders reviewed and provided feedback concerning the study design, data collection and analysis, decision to publish, or preparation of the manuscript.”

4. Thank you for stating the following in your Competing Interests section:

“None declared.”

Please complete your Competing Interests on the online submission form to state any Competing Interests. If you have no competing interests, please state "The authors have declared that no competing interests exist.", as detailed online in our guide for authors at http://journals.plos.org/plosone/s/submit-now

This information should be included in your cover letter; we will change the online submission form on your behalf.

Response: Thank you for the suggested text, we have added to the revised cover letter.

5. Thank you for stating the following in the Acknowledgments Section of your manuscript:

“We would like to thank the study participants who volunteered for the study. We also thank the Zambian Ministry of Health and the Lusaka Provincial Health Officer for their leadership on all facets of the national HIV programme. We would also like to thank the healthcare workers and CIDRZ research staff who made this study possible.”

We note that you have provided funding information that is not currently declared in your Funding Statement. However, funding information should not appear in the Acknowledgments section or other areas of your manuscript. We will only publish funding information present in the Funding Statement section of the online submission form.

Please remove any funding-related text from the manuscript and let us know how you would like to update your Funding Statement. Currently, your Funding Statement reads as follows:

“Funding for this work was provided through the Centers for Disease Control and Prevention (CDC) and the President’s Emergency Plan For AIDS Relief (PEPFAR) through grant number: NU2GGH001920.”

Please include your amended statements within your cover letter; we will change the online submission form on your behalf.

Response: Unsure where the overlap in funders and acknowledgements occurs – CDC/PEPFAR are different from Zambia Ministry of Health and CIDRZ.

6. Please note that your Data Availability Statement is currently missing the repository name and/or the DOI/accession number of each dataset OR a direct link to access each database. If your manuscript is accepted for publication, you will be asked to provide these details on a very short timeline. We therefore suggest that you provide this information now, though we will not hold up the peer review process if you are unable.

Response: Minimal data has been published through Data Dryad. We have amended the data availability statement as follows: “Minimal deidentified, aggregate data are available through Data Dryad (DOI: 10.5061/dryad.k98sf7mnf). Additional deidentified data may be made available upon reasonable request to the corresponding author.”

7. Please include captions for your Supporting Information files at the end of your manuscript, and update any in-text citations to match accordingly. Please see our Supporting Information guidelines for more information: http://journals.plos.org/plosone/s/supporting-information.

Response: Thank you, we have amended accordingly.

8. If the reviewer comments include a recommendation to cite specific previously published works, please review and evaluate these publications to determine whether they are relevant and should be cited. There is no requirement to cite these works unless the editor has indicated otherwise.

Response: Thank you for this clarification we have revised as we feel is valuable to improve the manuscript.

Additional Editor Comments:

To author team

Thanks for asking me about reviewer name suggestion.

Currently we are going with the available review reports.

Kindly submit atleast 2-3 potential reviewers' name which can review for the journal. Kindly keep in mind, that they should be from other institute/region and not be your recent past co-authors. It is not necessary that we will send your manuscript to them for review.

Thanks and regards

Best wishes

Response: Many thanks, we will be sure to provide reviewer recommendations with the revision for your consideration – understanding that they may not be approached for review.

***Review Comments to the Author***

Reviewer #1: The manuscript has valuable programmatic findings, particularly the feasibility of integrating RITA into routine services and the potential importance of behavioral risk assessment. However, the small RITA-recent sample, observational comparison, differential contact elicitation, limited facility number, and uncertainty surrounding attribution of social harms should be addressed before the conclusions can be considered sufficiently robust.

Response: Thank you for your feedback. We have made every effort to incorporate into the revised manuscript.

1) The study addresses an important and timely question regarding the integration of HIV recency testing into index testing services. However, the authors should clarify more explicitly what incremental benefit individual-level recency testing is expected to provide over existing risk-based index testing approaches.

Response: Thank you for this comment, we have added text in the background section to help set expectations of HIV recency testing.

Background: “As part of the study, we evaluated the effects of the recency testing strategy on contact positivity according to index recency status (i.e., recent versus long-term HIV acquisition) with the expectation that contact(s) of individuals identified as HIV recent may be at higher risk of HIV, and thus, have a different potential index testing yield.”

2) 20 of 317 participants (6.3%) were classified as RITA-recent, resulting in very small numbers for several key analyses. The authors should emphasize the limited statistical power and interpret the estimates, particularly the large prevalence ratios and wide confidence intervals, with appropriate caution.

Response: Thank you for this note. We have reported 95% confidence intervals to contextualize the data appropriately and been careful to not overstep the data constraints when reporting findings.

3) The observational design does not permit a definitive conclusion that recency testing “did not improve” index-testing efficiency. Since RITA-recent participants received additional partner elicitation whereas long-term participants did not, differences may reflect selection and intervention effects rather than recency status alone.

Response: Thank you for your note. In the data that we have analyzed we have found that there is not a significant difference between the long-term and recently infected yield. The limitations to the data have been reporting in the limitations section including the limited sample available testing RITA recent. We have made reference to the supplemental file that helps breakdown the findings per elicitation to improve clarity.

Results: “The proportion of newly positive index contacts among RITA-recent participants was higher for initial elicitation contacts compared to re-elicitation contacts at 30.0% and 5.0%, respectively (S1 Table).”

4) The comparison of HIV positivity among contacts should account more explicitly for differences in the number of contacts elicited and the proportion successfully tested between RITA-recent and long-term groups. The markedly smaller number of contacts in the recent group makes direct comparison potentially unstable.

Response: These are observational data being reported as (observationally) collected. There are potential differences in the recent and long-term populations here, as expected .

5) The manuscript describes a mixed-effects Poisson regression with facility-level random effects, but only two study facilities were included. The authors should justify the use and reliability of random-effects estimation with only two clusters or clarify whether facility clustering was handled differently in the relevant analyses.

Response: Thank you for this note. We have modified the methods section accordingly.

Methods: “Poisson regression to estimate prevalence of recency by participant sociodemographic and behavioral characteristics allowing for random effects at the facility level to account for potential facility level correlation.”

6) The association between condomless sex and recent HIV acquisition is striking but should not be interpreted as establishing a causal relationship. The authors should also discuss the possibility of recall and social-desirability bias in self-reported sexual behaviors, particularly given the sensitive nature of the questions.

Response: Thank you for this note. We agree, and have not made claims about a causal association between condomless sex and HIV recency.

7) The latent class analysis identifies two behavioral profiles, but the relatively small number of participants classified as RITA-recent limits the ability to establish a robust relationship between latent classes and recent infection. Additional justification for the selected number of classes and model fit would strengthen this analysis.

Response: Thank you for this note. We have modified the methods accordingly.

Methods: “This analysis considered variables of alcohol consumption, no condom use at last sex, transactional sex, and ever having sex while drunk or high on drugs. The number of latent classes was decided in the context of data limitations and the ability to detect meaningful differences across classes.”

8) The finding that 8.9% of participants reported social harm is important, but attribution specifically to recency testing remains uncertain because participants were newly diagnosed with HIV and also underwent partner notification and other post-diagnosis procedures. The Discussion appropriately acknowledges this issue, but the Abstract and Conclusion should avoid implying that these harms were necessarily caused by recency testing itself.

Response: Thank you for this note. We have modified the abstract accordingly.

Abstract, conclusions: “While integrating recency testing into routine index testing was feasible, our findings do not support its use to improve efficiency. Risk behavior assessment may help prioritize PLHIV for recency and index testing.”

9) The manuscript should discuss more explicitly that a RITA-recent classification represents an epidemiological estimate of recent infection rather than definitive determination of when or from whom HIV was acquired. This distinction is particularly important when individual-level results are returned and may influence perceptions regarding sexual partners or transmission.

Response: Thank you for this note. We have considered and feel it is adequately addressed.

10) The exclusion of populations considered at heightened risk of social harm, together with recruitment from only two urban facilities in Lusaka, substantially limits generalizability. The authors acknowledge this limitation, but it should be emphasized when proposing risk-based behavioral screening as an alternative strategy.

Response: Thank you for this note. We have outlined the population and tools thoroughly.

****End itemized review response

We would, again, like to thank the editor and reviewer for their time and thoughtful comments. We believe that edits made in response to the reviewer notes have further strengthened the manuscript and we look forward to receiving your feedback in due course.

Kind Regards,

Jake

# Peer review: Decision Letter 1

**DOI:** 10.1371/journal.pone.0361598.r003

**Authors:** Sahjid Mukhida

13 Oct 2026

Results of integrating recency testing into routine index testing services in Lusaka, Zambia: A mixed effects regression analysis

PONE-D-26-20164R1

Dear Dr. Pry,

We’re pleased to inform you that your manuscript has been judged scientifically suitable for publication and will be formally accepted for publication once it meets all outstanding technical requirements.

Within one week, you’ll receive an e-mail detailing the required amendments. When these have been addressed, you’ll receive a formal acceptance letter and your manuscript will be scheduled for publication.

An invoice will be generated when your article is formally accepted. Please note, if your institution has a publishing partnership with PLOS and your article meets the relevant criteria, all or part of your publication costs will be covered. Please make sure your user information is up-to-date by logging into Editorial Manager at Editorial Manager® and clicking the ‘Update My Information' link at the top of the page. For questions related to billing, please contact billing support.

If your institution or institutions have a press office, please notify them about your upcoming paper to help maximize its impact. If they’ll be preparing press materials, please inform our press team as soon as possible -- no later than 48 hours after receiving the formal acceptance. Your manuscript will remain under strict press embargo until 2 pm Eastern Time on the date of publication. For more information, please contact onepress@plos.org.

Kind regards,

Sahjid Mukhida

Academic Editor

PLOS One

Additional Editor Comments (optional):

To Author team

First I have to appreciate your efforts for writing and revising manuscript. We are appologise to convey the reviwer's report.

As per the available reports, we can accept your manuscript for publication in current form

Best wishes

Reviewers' comments:

Reviewer's Responses to Questions

**Comments to the Author**

Reviewer #1: All comments have been addressed

**********

2. Is the manuscript technically sound, and do the data support the conclusions?

Reviewer #1: Yes

**********

3. Has the statistical analysis been performed appropriately and rigorously?

Reviewer #1: Yes

**********

4. Have the authors made all data underlying the findings in their manuscript fully available?

The PLOS Data policy

Reviewer #1: Yes

**********

5. Is the manuscript presented in an intelligible fashion and written in standard English?

Reviewer #1: Yes

**********

Reviewer #1: I have gone through the whole manuscript and author response.

I have satisfied with the author response

Recommending for publication

**********

what does this mean?). If published, this will include your full peer review and any attached files.

If you choose “no”, your identity will remain anonymous but your review may still be made public.

**Do you want your identity to be public for this peer review?**  For information about this choice, including consent withdrawal, please see our Privacy Policy

Reviewer #1: **Yes:**  Dr Nikunja Kumar Das TV

**********

# Editor report: Acceptance letter

**DOI:** 10.1371/journal.pone.0361598.r004

**Authors:** Sahjid Mukhida

PONE-D-26-20164R1

PLOS One

Dear Dr. Pry,

I'm pleased to inform you that your manuscript has been deemed suitable for publication in PLOS One. Congratulations! Your manuscript is now being handed over to our production team.

At this stage, our production department will prepare your paper for publication. This includes ensuring the following:

* All references, tables, and figures are properly cited

* All relevant supporting information is included in the manuscript submission,

* There are no issues that prevent the paper from being properly typeset

You will receive further instructions from the production team, including instructions on how to review your proof when it is ready. Please keep in mind that we are working through a large volume of accepted articles, so please give us a few days to review your paper and let you know the next and final steps.

Lastly, if your institution or institutions have a press office, please let them know about your upcoming paper now to help maximize its impact. If they'll be preparing press materials, please inform our press team within the next 48 hours. Your manuscript will remain under strict press embargo until 2 pm Eastern Time on the date of publication. For more information, please contact onepress@plos.org.

You will receive an invoice from PLOS for your publication fee after your manuscript has reached the completed accept phase. If you receive an email requesting payment before acceptance or for any other service, this may be a phishing scheme. Learn how to identify phishing emails and protect your accounts at https://explore.plos.org/phishing.

If we can help with anything else, please email us at customercare@plos.org.

Thank you for submitting your work to PLOS One and supporting open access.

Kind regards,

PLOS One Editorial Office Staff

on behalf of

Dr. Sahjid Mukhida

Academic Editor

PLOS One
